# Anti-Inflammatory Activity of *Pleurotus ostreatus*, a Culinary Medicinal Mushroom, in Wistar Rats

**DOI:** 10.1155/2020/6845383

**Published:** 2020-03-05

**Authors:** W. J. A. Banukie N. Jayasuriya, Shiroma M. Handunnetti, Chandanie A. Wanigatunge, Gita H. Fernando, D. Thusitha U. Abeytunga, T. Sugandhika Suresh

**Affiliations:** ^1^Department of Pharmacy and Pharmaceutical Sciences, Faculty of Allied Health Sciences, University of Sri Jayewardenepura, Nugegoda, Sri Lanka; ^2^Institute of Biochemistry, Molecular Biology and Biotechnology, University of Colombo, Colombo, Sri Lanka; ^3^Department of Pharmacology, Faculty of Medical Sciences, University of Sri Jayewardenepura, Nugegoda, Sri Lanka; ^4^Department of Chemistry, Faculty of Science, University of Colombo, Colombo, Sri Lanka; ^5^Department of Biochemistry, Faculty of Medical Sciences, University of Sri Jayewardenepura, Nugegoda, Sri Lanka

## Abstract

*Context*. *Pleurotus ostreatus* (*P.o*) is a culinary mushroom which is commonly called as “oyster mushroom” belonging to the Basidiomycetous fungi of the order Agaricales and family Pleurotaceae.

**Objectives:**

The present study investigates the anti-inflammatory potential of *P.o*) is a culinary mushroom which is commonly called as “oyster mushroom” belonging to the Basidiomycetous fungi of the order Agaricales and family Pleurotaceae. *Materials and Methods*. Anti-inflammatory activity was evaluated using suspensions of freeze-dried and powdered (SFDP) *P.o*) is a culinary mushroom which is commonly called as “oyster mushroom” belonging to the Basidiomycetous fungi of the order Agaricales and family Pleurotaceae. *P.o*) is a culinary mushroom which is commonly called as “oyster mushroom” belonging to the Basidiomycetous fungi of the order Agaricales and family Pleurotaceae. *P.o*) is a culinary mushroom which is commonly called as “oyster mushroom” belonging to the Basidiomycetous fungi of the order Agaricales and family Pleurotaceae. *in vivo* and *in vitro* assays.

**Results:**

At doses of 500–1000 mg/kg, the SFDP of *P.o*) is a culinary mushroom which is commonly called as “oyster mushroom” belonging to the Basidiomycetous fungi of the order Agaricales and family Pleurotaceae. *P.o*) is a culinary mushroom which is commonly called as “oyster mushroom” belonging to the Basidiomycetous fungi of the order Agaricales and family Pleurotaceae. *P.o*) is a culinary mushroom which is commonly called as “oyster mushroom” belonging to the Basidiomycetous fungi of the order Agaricales and family Pleurotaceae. *P.o*) is a culinary mushroom which is commonly called as “oyster mushroom” belonging to the Basidiomycetous fungi of the order Agaricales and family Pleurotaceae. *in vitro* assays. *P* < 0.05). Dose-dependent inhibition of NO production was seen with *in vitro* assays. *P.o*) is a culinary mushroom which is commonly called as “oyster mushroom” belonging to the Basidiomycetous fungi of the order Agaricales and family Pleurotaceae. *r* = 0.95; *P* < 0.05). Dose-dependent inhibition of NO production was seen with *Discussion and Conclusion.* The promising activity of culinary mushroom *P.o* against inflammation suggests its potential application as a functional food during inflammatory conditions.*P.o*) is a culinary mushroom which is commonly called as “oyster mushroom” belonging to the Basidiomycetous fungi of the order Agaricales and family Pleurotaceae.

## 1. Introduction

Inflammation is a protective response to tissue injury which usually subsides after healing occurs [1]. Inflammation can be triggered by an innocuous agent or by an autoimmune process, as in rheumatoid arthritis [1]. During the process of inflammation, proinflammatory cells (macrophages and monocytes) release proinflammatory mediators, such as tumour necrosis factor-*α* (TNF-*α*), cytokines (interleukin (IL)-1 and IL-2), prostaglandin E_2_, and nitric oxide (NO) [2].

Increased expression and activity of cyclooxygenase (COX)-2 and inducible nitric oxide synthase (iNOS) also play an important role in the inflammatory process [3]. In such cases, the defense reactions may cause tissue injury where anti-inflammatory drugs may be needed to overcome the inflammatory process. Currently available anti-inflammatory drugs possess side effects which can cause problems during clinical use [4]. Nonsteroidal anti-inflammatory drugs (NSAIDs) which are COX-2 inhibitors are known to cause serious gastrointestinal disturbances including bleeding, ulceration, and other effects such as hypersensitivity reactions [2]. This has led to an increase in demand for natural products with anti-inflammatory activity with fewer side effects. Plants as well as mushrooms have been targeted in the search for new anti-inflammatory agents [5]. Some plants and mushrooms have been screened and identified as sources to treat inflammation [6, 7].

*Pleurotus* species which are commonly called “oyster mushrooms” belong to the Basidiomycetes fungi of the order Agaricales and family Pleurotaceae. They are characterized by a fruiting body with an eccentric stalk and a wide cap [8]. *Pleurotus ostreatus* is an edible mushroom commonly known as American oyster.

Studies have reported various properties of *P.o* such as antinociceptive [9], hypocholesterolaemic [10], antioxidant [11], and antitumour effects [12]. Our recent investigations also showed the oral hypoglycaemic property of *P.o* and its probable mechanism of action [13, 14]. We have also demonstrated the value of *P.o* as a functional food in diabetes mellitus [14].

Previous studies have reported [15] anti-inflammatory effects of *P. eous* [5], *P*. *pulmonarius* [6], *P. sajor-caju* [16], *P. florida* [17], *P. eryngii* [18], and *P. citrinopileatus* [19]. Bobek and Galbavy [20] studied the anti-inflammatory activity of *P.o* in an acute colitis-induced rat model while Rivero-Pérez et al. [21] evaluated the anti-inflammatory activity of *P.o* in mouse ears treated with 12-O-tetradecanoylphorbol-13-acetate. Jedinak et al. [22] reported the anti-inflammatory mechanisms of *P.o* using lipopolysaccharide-stimulated RAW264.7 macrophages. However, anti-inflammatory properties and underlying molecular mechanisms of *P.o* using a carrageenan-stimulated inflammatory model have not been addressed.

Therefore, the purpose of this study was to investigate the anti-inflammatory potential of *P.o* using carrageenan-induced rat paw oedema model and to identify the possible mechanisms underlying the activity. Effectiveness of *P.o* in the treatment of inflammatory pathologies in diabetic rats was also evaluated for the first time.

## 2. Methods

### 2.1. Study Setting

The study was conducted at the Animal House and Department of Biochemistry, Faculty of Medical Sciences, University of Sri Jayewardenepura, Sri Lanka, and the Institute of Biochemistry, Molecular Biology and Biotechnology, University of Colombo, Sri Lanka. Ethical clearance was granted by the Ethics Review Committee of the Faculty of Medical Sciences, University of Sri Jayewardenepura, Sri Lanka (no. 380/8).

### 2.2. Experimental Animals

Healthy adult Wistar rats (200–250 g) were purchased from the Medical Research Institute Colombo. They were housed under standardized animal house conditions and had access to food (WHO recommended food formula: maize 40.1 kg, broken brown rice 10 kg, rice bran 2.5 kg, wheat bran 2 kg, wheat flour 13.5 kg, fish meal 8 kg, soya meal 8 kg, sugar 2.5 kg, soya oil 2 kg, grass powder 3 kg, bone meal 1.5 kg, mineral mix 0.4 kg, vitamin mix 0.24 kg, NaCl 0.2 kg, beta mix E50 0.02 kg, DL methionine 0.05 kg, milk powder 6 spoons, and vitamin B complex 600 tablets/100 kg) and water *ad libitum*.

### 2.3. Collection and Preparation of Mushrooms

Fresh *P.o* grown using the spawn provided by the Mushroom Cultivation Centre, Export Research Board (Ratmalana, Sri Lanka), were collected from a local farm. The identification and authentication were done by studying the spore print and the shape of the cap (fan-shaped) and the stipe (eccentric). Fresh *P.o* (1 kg) was washed with water to remove soil particles, freeze-dried (Eyela, FD-5N, Japan), and ground with a commercial blender (Sonica, SA-317, China). The SFDP *P.o* were freshly prepared with distilled water (DW) prior to the feeding of rats.

### 2.4. Extraction of Mushroom

Fresh mushroom (3.0 kg) was homogenized with a homogenizer (Ultra-Turrax®, T 25 basic, IKA-Werke, Germany) and left overnight soaked in 1.5 L of distilled acetone. The solution was removed and again extracted using 1.5 L of acetone according to the same method stated. The solution was filtered using a filter paper to remove particles. The extract was concentrated using a rotary evaporator (Eyela, N-N series, Japan) and freeze-dried (FD-5N, Eyela, Japan). The dried material (65 g) was stored in a refrigerator. The AE of *P.o* was dissolved in DW by sonication prior to feeding to rats.

### 2.5. Anti-Inflammatory Activity

#### 2.5.1. Anti-Inflammatory Activity of *P. ostreatus* in Carrageenan-Induced Paw Oedema in Healthy Wistar Rats

The anti-inflammatory activity of *P.o* was determined using the carrageenan-induced paw oedema model [23]. Healthy, male, Wistar rats (*n* = 48) were selected and the volume of the left hind paw of each rat was measured using a plethysmometer (*V*_o_) (Panlab s.I., Barcelona, Spain). These rats were randomly divided into eight equal groups and treated orally in the following manner. Groups 1, 2, 3, 4, and 5 were orally administered with five different doses of SFDP *P.o*, 125 mg/kg, 250 mg/kg, 500 mg/kg, 750 mg/kg, and 1000 mg/kg, respectively. Group 6 was given AE of *P.o* at a dose of 500 mg/kg. Group 7 was treated with the reference drug, indomethacin (10 mg/kg), whereas rats in group 8 received 2.5 mL of distilled water and served as the control group. After 1 hour, 0.1 mL of 1% carrageenan suspension in normal sterile saline was injected subcutaneously into the plantar surface of the left hind paw of rats under mild ether anaesthesia. The left hind paw volumes of these rats were measured at hourly intervals up to 5^th^ hour (*V*_t_). Using these volumes, increase in paw oedema and percent inhibition of paw oedema were calculated as follows:

(1) $$\begin{array}{c} \%\text{ inhibition of oedema}=\frac{\left(V_{\text{t}}-V_{0}\right)-{\left(V_{\text{t}}-V_{0}\right)}_{\text{treated}}\times 100}{{\left(V_{\text{t}}-V_{0}\right)}_{\text{control}}}. \end{array}$$

#### 2.5.2. Anti-Inflammatory Activity of *P. ostreatus* in Carrageenan-Induced Paw Oedema in Alloxan-Induced, Diabetic Wistar Rats

The rats were injected with alloxan monohydrate dissolved in normal sterile saline at a dose of 40 mg/kg intravenously to induce diabetes. After 72 hours, the rats (*n* = 30) showing fasting serum glucose levels above 180 mg/dL were selected for the study. Anti-inflammatory activity of SFDP *P.o* (doses used: 500 mg/kg and 1000 mg/kg), AE of *P.o* (500 mg/kg), and indomethacin (10 mg/kg) were determined according to the method described above. The group receiving 2.5 mL of DW served as the control group. The mid- and high doses of SFDP *P.o*, 500 mg/kg and 1000 mg/kg, were selected from the study conducted with healthy rats.

### 2.6. Mechanisms of Anti-Inflammatory Activity

#### 2.6.1. Antihistamine Activity

Healthy, male, Wistar rats (*n* = 24) were selected, and fur on the left posterior lateral side was removed by gentle shaving. Rats were randomly assigned to three groups after 24 hours and treated orally in the following manner. Group 1: 500 mg/kg of AE of *P.o*; Group 2: 0.67 mg/kg of chlorpheniramine; and Group 3: 2.5 mL of DW. After 1 hour, 50 *μ*L of 200 *μ*g/mL of histamine dihydrochloride in normal sterile saline was subcutaneously injected into the shaved area of the skin, and the area of the wheal formed was determined after 2 minutes [24].

#### 2.6.2. Assay for Carrageenan-Induced Infiltration of Rat Peritoneal Cells

Three groups of rats (*n* = 18) were orally treated with 500 mg/kg of AE of *P.o*, 10 mg/kg of prednisolone, and DW. After 1 hour, 1 mg/mL of carrageenan in normal sterile saline was injected intraperitoneally at a dose of 5 mg/kg under ether anaesthesia. After 2 hours, 40 mL of sterile phosphate-buffered saline (PBS) with a pH of 7.4 was injected into the peritoneal cavity. Five minutes later, 35 mL of peritoneal fluid was drained using an 18 G cannula and centrifuged at 150*g* for 10 minutes at 4°C. The peritoneal cells were resuspended in 1 mL of PBS after removing the supernatant. A 50 *μ*L cell suspension was mixed with 10 *μ*L of 1% neutral red. Phagocytic/macrophage cell counts were made using a haemocytometer [24].

#### 2.6.3. *In Vivo* Assay for Nitric Oxide Production by Peritoneal Cells

Three groups of rats (*n* = 18) were treated with 500 mg/kg of AE of *P.o*, 10 mg/kg of prednisolone, and DW. Peritoneal cells exposed to extracts *in vivo* were harvested from rats orally treated with extract as described above. The *in vivo* NO inhibitory effect of the AE of *P.o* and prednisolone was studied according to the method described by Handunnetti et al. [24]. The peritoneal cells collected from each rat were plated in 96-well tissue culture plates at 1 × 10^6^ cells/mL in RPMI 1640 medium (GIBCO BRL, Life Technologies, Scotland) supplemented with 1% bovine serum albumin (BSA) (Sigma Chemicals Company, St Louis, Mo, USA) and incubated at 37°C in 5% CO_2_ incubator (Sanyo Electric. Co. Ltd., Osaka, Japan). The culture supernatant was aspirated from each well, after 24 hours and then centrifuged at 10000*g* for 10 minutes and the clear supernatant was obtained. In order to measure the NO concentration, 100 *μ*L of culture supernatant was mixed with an equal volume of Griess reagent (mixture of equal proportion of 1% sulphanilamide in 5% phosphoric acid and 0.1% n-(1-naphthyl) ethylenediamine hydrochloride in DW) and incubated at 25°C for 15 minutes and optical density (OD) was read at 540 nm in an ELISA plate reader (ELX 800, Bio-Tek Instruments Inc, USA). The NO concentration was calculated using a calibration curve between 0.195 and 100 *μ*M NaNO_2_.

#### 2.6.4. *In Vitro* Assay for Nitric Oxide Production by Peritoneal Cells

The *in vitro* NO inhibitory effect was studied according to the method described by Maccioni et al. [25]. The viability of peritoneal cells after 30 minutes of incubation with different concentrations of AE of *P.o* was assessed by Trypan blue exclusion test [24]. Peritoneal cells harvested from rats administered with carrageenan (5 mg/kg) intraperitoneally were placed in 96-well tissue culture plate. The cells were treated *in vitro* with 3.90–125 *μ*g/mL of AE of *P.o* in RPMI 1640 medium supplemented with 1% BSA at 37°C in a 5% CO_2_ incubator for 30 minutes. In addition, peritoneal cells were treated with RPMI 1640 medium as a negative control. Cells were centrifuged at 150*g* for 2 minutes and resuspended in culture medium containing 1% BSA and cultured for 24 hours after which the culture supernatant was aspirated. The culture supernatant was aspirated from each well, after 24 hours. It was then centrifuged at 10000*g* for 10 minutes, to obtain a clear supernatant, and the NO concentration was measured.

#### 2.6.5. Assay for Membrane Stabilizing Activity

This assay was performed using heat-induced haemolysis of rat erythrocytes. A ten-fold dilution series ranging from 0.001 to 1000 *μ*g/mL of AE of *P.o* and aspirin (*n* = 6 each) was made using PBS. PBS was used as a control. Vials containing 20 *μ*L of rat blood and different concentrations of AE of *P.o*, aspirin, and PBS (1 mL each) were prepared in triplicate. The vials were incubated at 37°C for 15 minutes and centrifuged at 1500*g* for 3 minutes. The supernatants were removed and the cells were resuspended in 1 mL of PBS. The vials were then incubated at 54°C for 25 minutes, centrifuged at 1500*g* for 5 minutes, and supernatants were obtained (200 *μ*L); OD value was measured at 540 nm. Percentage inhibition of haemolysis was calculated with respect to the controls:

(2) $$\begin{array}{c} \text{percent inhibition of haemolysis}=\left[\frac{{\left(\text{OD}\right)}_{\text{control}}-{\left(\text{OD}\right)}_{\text{sample}}}{{\left(\text{OD}\right)}_{\text{control}}}\right]\;\times 100. \end{array}$$

### 2.7. Statistical Analysis

Results were presented as mean ± SEM. The results were analyzed for statistical significance using Student's *t*-test. Effects of different doses were analyzed by analysis of variance (ANOVA). Statistical analysis was done using Microsoft Excel 2007 version and SPSS 17. *P* values <0.05 were considered as significant.

## 3. Results

### 3.1. Anti-Inflammatory Activity of *P. ostreatus* in Carrageenan-Induced Paw Oedema in Healthy Wistar Rats

**Figure 1:**
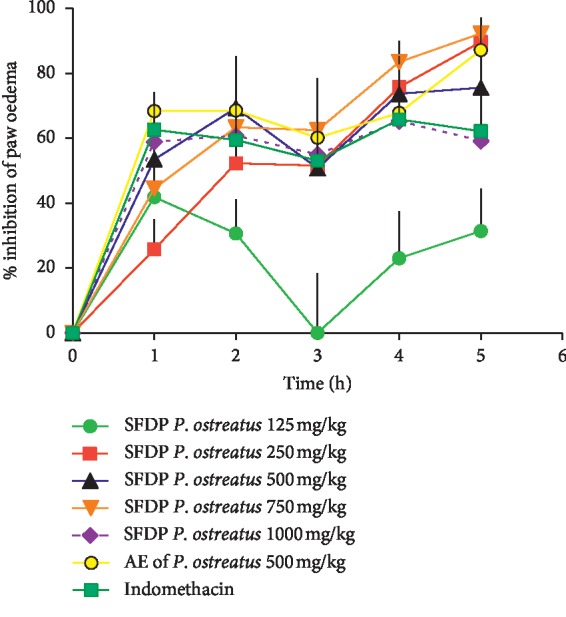
Inhibition of carrageenan-induced rat paw oedema, following oral administration of different doses of suspensions of freeze-dried and powdered *P. ostreatus*, acetone extract of *P. ostreatus*, and indomethacin using healthy rats. Values are expressed as mean ± SEM.

The results of anti-inflammatory activity of *P.o* in healthy Wistar rats are shown in Figure 1. When compared to the control group, treatment with SFDP *P.o* at doses of 250–1000 mg/kg and indomethacin showed significant (*P* < 0.05) inhibition of rat paw oedema. The low dose (125 mg/kg) did not significantly (*P* > 0.05) impair paw oedema at 2–5 hours whereas the reference drug indomethacin showed significant inhibition up to the 5^th^ hour. The SFDP *P.o* at doses of 500–1000 mg/kg showed long-lasting activity at both early and late phases. The SFDP *P.o* at 750 mg/kg showed maximum inhibition of oedema of 92% (after 5 hours) (*P* < 0.05). The inhibition pattern of the highest dose of SFDP *P.o* (1000 mg/kg) was similar to that of indomethacin (*P* < 0.05). However, according to Figure 1, the inhibition of carrageenan-induced paw oedema by SFDP *P.o* was not dose-dependent. The AE of *P.o* showed maximum inhibition of oedema of 87% (*P* < 0.01).

### 3.2. Anti-Inflammatory Activity of *P. ostreatus* in Carrageenan-Induced Paw Oedema in Alloxan-Induced, Diabetic Wistar Rats

**Figure 2:**
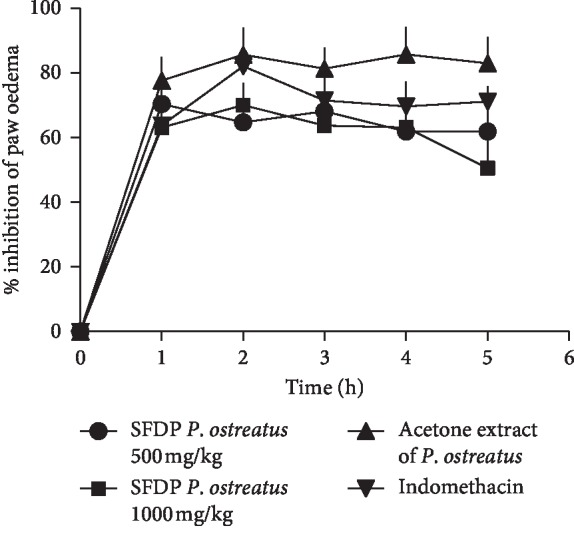
Inhibition of carrageenan-induced rat paw oedema, following oral administration of different doses of suspensions of freeze-dried and powdered *P. ostreatus* and acetone extract of *P. ostreatus* using alloxan-induced diabetic rats. Values are expressed as mean ± SEM.

The results are summarized in Figure 2. Treatment with SFDP *P.o* at doses of 500 mg/kg (up to 5^th^ hour) and 1000 mg/kg (up to 4^th^ hour) showed significant (*P* < 0.05) inhibition of paw oedema in diabetic rats when compared to the diabetic control group. The AE of *P.o* (500 mg/kg) showed significant (*P* < 0.01) inhibition of paw oedema in diabetic rats after 1–5 hours. The anti-inflammatory effect exerted by the SFDP *P.o* at doses of 500 mg/kg and 1000 mg/kg and AE of *P.o* was comparable to indomethacin (*P* > 0.05). The SFDP *P.o* at doses of 500 mg/kg (after 1^st^ hour) and 1000 mg/kg (after 2^nd^ hours) showed maximum inhibition of oedema of 70% whereas the AE of *P.o* showed maximum inhibition of oedema of 86% (*P* < 0.01). The dose of 1000 mg/kg showed highest activity at an early phase of carrageenan-induced paw oedema whereas the dose of 500 mg/kg showed long-lasting activity at both early and late phases. However, according to Figure 2, the inhibition of carrageenan-induced paw oedema in alloxan-induced, diabetic Wistar rats by SFDP *P.o* was not dose-dependent.

### 3.3. Antihistamine Activity

Compared to the control, treatment with AE of *P.o* and chlorpheniramine significantly reduced (*P* < 0.0001) the area of wheal formed (52.1 ± 1.1% and 57.9 ± 1.5%, respectively) on the skin by the injection of histamine. However, the antihistamine effect exerted by AE of *P.o* was less than the effect exerted by chlorpheniramine.

### 3.4. Inhibition of Carrageenan-Induced Infiltration of Rat Peritoneal Cells

**Figure 3:**
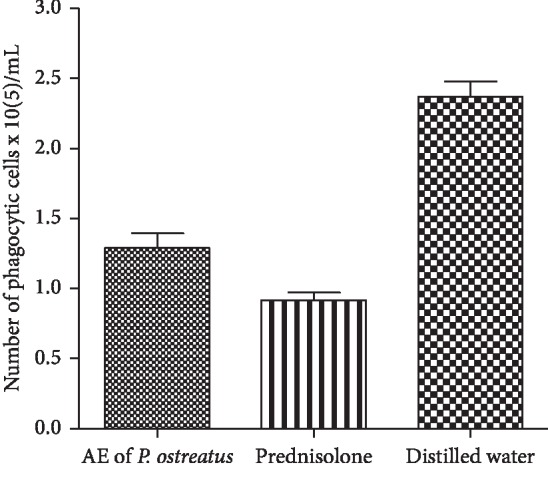
Effect of acetone extract of *P. ostreatus* on rat peritoneal cell infiltration. Values are expressed as mean ± SEM.

The AE of *P.o* and prednisolone significantly inhibited the number of phagocytic cells infiltrating into the peritoneal cavity when compared to the control group ((*P* < 0.01), (Figure 3)).

### 3.5. Inhibition of Nitric Oxide Production by Peritoneal Cells following *In Vivo* Treatment

Peritoneal cells harvested from rats after oral administration of AE of *P.o* and prednisolone significantly inhibited NO production when compared with the control group (91.2 ± 1.3% and 95.6 ± 0.2% reduction; *P* < 0.001, respectively).

### 3.6. Inhibition of Nitric Oxide Production by Peritoneal Cells following *In Vitro* Treatment

**Figure 4:**
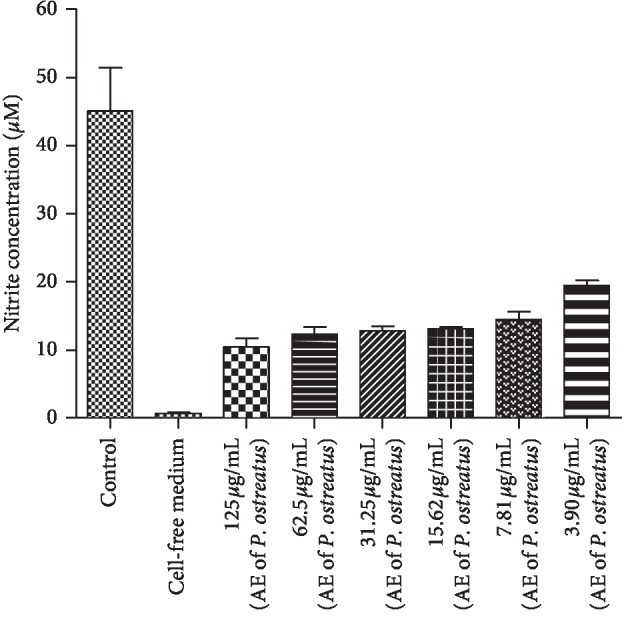
Effect of different concentrations of acetone extract of *P. ostreatus* on NO production by rat peritoneal cells. Values are expressed as mean ± SEM.

Viable cell counts obtained after 30 minutes of incubation with different concentrations of AE of *P.o* showed concentrations of 125 *μ*g/mL (72.7%) or lower, were not cytotoxic to peritoneal cells, and were suitable for the *in vitro* treatment of assay for NO inhibitory activity. Treatment of peritoneal cells with 3.90–125 *μ*g/mL of AE of *P.o in vitro* significantly inhibited the NO production (*P* < 0.01). Apart from the lowest dose (3.90 *μ*g/mL), other tested doses of AE of *P.o* demonstrated a dose-dependent inhibition of NO production (*r* = 0.95; *P* < 0.05). Maximum inhibition was observed at a dose of 125 *μ*g/mL ((76.9 ± 2.9%) (Figure 4)).

### 3.7. Membrane Stabilizing Activity

Apart from the lowest concentration of AE of *P.o* (0.001 *μ*g/mL), other higher concentrations significantly inhibited the heat-induced haemolysis of rat erythrocytes *in vitro*. The highest inhibitory activity (52.6 ± 2.1%.) was shown by the dose of 100 *μ*g/mL. Decrease of the inhibition of haemolysis at 1000 *μ*g/mL may be due to the multicomponent nature of the AE of *P.o*, which could contain active compounds with different effects. Hence, it can be speculated that at the higher dose levels, anti-inflammatory activity might have been masked by the presence of the opposite effect producing active compounds. This type of dose-independent membrane stabilizing activity is reported by various studies on medicinal plants [26, 27].

**Figure 5:**
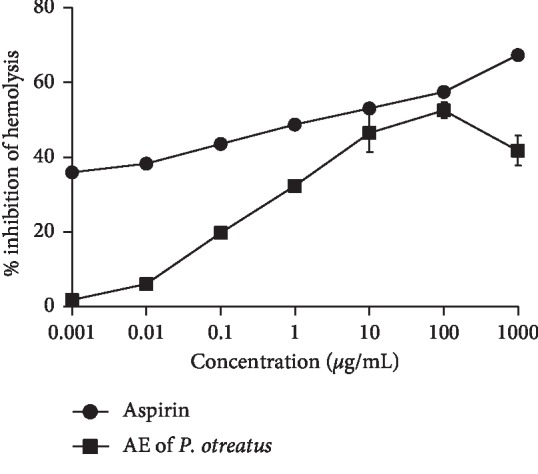
Percentage inhibition of heat-induced haemolysis of rat erythrocytes by acetone extract of *P. ostreatus*. Values are expressed as mean ± SEM.

With the increasing concentrations of aspirin, the inhibition of haemolysis increased dose-dependently (*r* = 0.76; *P* < 0.05) (Figure 5) according to the Pearson correlation.

## 4. Discussion

In the present study, an attempt has been made to evaluate the anti-inflammatory activity of *P.o* by use of the carrageenan-induced paw oedema model. Inhibition of carrageenan-induced inflammation has played a pivotal role in the development of NSAIDs and COX inhibitors [28]. The inflammatory response of carrageenan-induced paw oedema test is biphasic with a maintenance phase in between (2-3 hours). The initial inflammatory phase is mediated by histamine and serotonin and increase in PG synthesis at the damaged tissue and lasting up to 2 hours, whereas the delayed inflammatory phase is mediated by leukotrienes, phagocytic cells, polymorphonuclear cells, monocytes, macrophages, PGs produced by tissue macrophages, oxygen free radicals, and NO and observed from 3 to 5 hours. During the maintenance phase, kinin-like substances, such as bradykinin are predominant [28].

Oral administration of a single dose of SFDP *P.o* and AE of *P.o* exerted significant anti-inflammatory activity in normal rats. When considering the dose study (Figure 1), it was noted that the SFDP *P.o* at doses of 250–1000 mg/kg had exerted a significant inhibition of rat paw oedema. However, the inhibition of carrageenan-induced paw oedema by SFDP *P.o* was not dose-dependent. This is probably due to the multicomponent nature of the *P.o* suspensions, which could contain active compounds with different effects. Hence, it can be speculated that at the higher dose levels, anti-inflammatory activity might have been masked by the presence of the opposite effect producing active compounds. This type of dose-independent anti-inflammatory activity is reported by various studies on medicinal plants [29] and the drug tacrolimus [30].

The AE of *P.o* (500 mg/kg) showed maximum inhibition of 87% (*P* < 0.01), at late phase of inflammation. Hence, the anti-inflammatory activity-guided fractionation of AE of *P.o* could be beneficial in the search of anti-inflammatory active agents.

The role of inflammation in diabetes has been extensively studied [31]. It has been reported that there is an increase in levels of cytokines in diabetic conditions [32]. Furthermore, diabetes results in poor anti-inflammatory capabilities [33]. Even though the efficacy of plants for the treatment of inflammatory conditions seen with diabetes has been reported, [34] studies using mushrooms are not documented.

Treatment with SFDP *P.o* at doses of 500 mg/kg and 1000 mg/kg and AE of *P.o* showed significant (*P* < 0.05) inhibition of paw oedema in diabetic rats which was comparable with indomethacin. Furthermore, the anti-inflammatory activity in diabetic rats, at doses of 500 mg/kg and 1000 mg/kg of SFDP *P.o*, and in AE of *P.o* (500 mg/kg) was higher than in healthy rats at early phase of carrageenan-induced paw oedema. Since the diabetes results in poor anti-inflammatory capabilities, this study supports the capability of *P.o* in the treatment of inflammatory pathologies in rats with diabetes. Similar effect was reported by Hassanabad et al. [34]. In the present study, carrageenan-induced paw oedema in the diabetic control group was higher than in healthy control rats after 2–5 hours. Similar to the observation by Hassanabad and coworkers [34], in our study too, there was a significant increase in inflammation of diabetic mice in the control group when compared with normal mice.

The SFDP *P.o* and AE of *P.o* impaired both the initial and late phases of carrageenan-induced inflammation. This study exhibited marked antihistamine activity of AE of *P.o* (52.1% at the dose of 500 mg/kg), and Vasudewa et al. [9] reported the antihistamine activity of SFDP *P.o* (50.7% at the dose of 1000 mg/kg) when evaluated by the wheal test. Histamine released from mast cells increases vascular permeability which is one of the essential features of the acute inflammatory response [1]. Therefore, initial phase inhibition could be partly due to the impairment of microvascular leakage by antihistamine activity.

Cyclooxygenase is the enzyme that catalyzes the first two steps in the biosynthesis of the PGs from the substrate arachidonic acid [2]. There are two isoforms of this enzyme, COX-1 and COX-2. Different classes of compounds are known to produce anti-inflammatory activity through the inhibition of COX-2 and/or iNOS activity and/or expression [3, 35]. COX inhibitory compounds have been isolated from mushrooms such as *Grifola frondosa* [36]. Glycoprotein isolated from *P. citrinopileatus* and water extract of *P.o* showed inhibition of LPS-induced production of PGE2 and NO through the downregulation of expression of COX-2 and iNOS in macrophages, respectively [19, 22]. Therefore, in the present study inhibition of the late phase of carrageenan-induced paw oedema by *P.o* may be attributed to COX inhibition and prostaglandin synthesis. Furthermore, *P.o* showed marked and dose-dependent antioxidant activity as observed by Vasudewa et al. [9], which can be linked to its anti-inflammatory action in the late phase.

Phagocytic cell migration to the site of inflammation is well known [1]. Nacife et al. [37] reported carrageenan-induced leukocyte migration. This model has been used to demonstrate the inhibition of peritoneal cell migration by *Ixora coccinea* [24]. This model in turn can be used to study the cell migration process and the inhibitory effects of drugs on cell migration. In the present study, the AE of *P.o* significantly reduced the migration of phagocytic cells in response to carrageenan-induced inflammatory stimulus. Hence, *in vivo* peritoneal infiltration assay emphasized another mechanism responsible for anti-inflammatory activity of *P.o*.

Nitric oxide is produced in cells constitutively or induced by various cell activators through the oxidation of L-arginine by isoenzymes (nitric oxide synthases). Expression of iNOS, one of the isoforms, generates higher levels of NO which contributes to the immune defense [3].

The NO generated diffuses to the vascular smooth muscle, activates soluble guanylate cyclase, leading to increased intracellular cGMP levels, and promotes relaxation of the smooth muscle causing vasodilatation [3]. Increased permeability of blood vessels leads to the exudation of plasma proteins and fluids into the tissues forming oedema [38].

In this study, treatment with AE of *P.o in vitro* and *in vivo* significantly inhibited NO production by rat peritoneal cells. The results of *in vitro* assay show that carrageenan induced an increase in NO production in peritoneal cells which was significantly (*P* < 0.01) reduced when treated with different concentrations of AE of *P.o*. This study suggests that AE of *P.o* is an effective inhibitor against carrageenan-induced NO production. Hence, inhibition of NO is likely to be another mechanism via *P.o*-induced anti-inflammatory activity in the late phase of the carrageenan-induced paw oedema test. Inhibition of NO production has been observed in other *Pleurotus* species as well. Chen et al. [19] reported the ability of *P. citrinopileatus* in inhibiting lipopolysaccharide-induced NO production *in vitro*.

Moreover, apart from the lowest dose (3.90 *μ*g/mL), other tested doses of AE of *P.o* demonstrated a dose-dependent inhibition of NO production in the present study. This suggests that the concentration of active constituents which are responsible for reducing NO production in AE of *P.o* is increased when increasing the dose. Methanol extract of *C. pruinosa* also inhibited NO production in LPS-stimulated RAW264.7 cells in a dose-dependent manner [39].

In addition, AE of *P.o* showed membrane stabilizing activity in the heat-induced haemolysis of rat erythrocytes *in vitro*. On exposure to hypotonic medium, heat, and phenylhydrazine may cause the lysis of the red blood cell (RBC) membrane which leads to haemolysis and oxidation of haemoglobin [40]. Red blood cell membranes are similar to lysosomal membrane components [7]. Thus, the inhibition of heat-induced RBC membrane lysis was considered as one of the mechanisms of anti-inflammatory activity of *P.o*. The AE of *P.o* may stabilize the lysosomal membrane, preventing the release of inflammatory mediators and lytic enzymes. Compounds which demonstrate membrane stabilization activity inhibit early phase of inflammatory reactions by preventing the release of phospholipases [41]. Hence, the membrane stabilization potential of AE of *P.o* can be attributed as another mechanism of the anti-inflammatory activity in early phase of carrageenan-induced paw oedema.

In the present study, we have conducted four experiments to study the mechanism of action of anti-inflammatory activity produced by *P.o*. In addition to these specific mechanisms, several other mechanisms may account for anti-inflammatory activity of *P.o*. Planas et al. [42] evaluated the opioid receptor-mediated activities on suppression of the inflammation in the carrageenan-induced paw oedema. The SFDP *P.o* and AE of *P.o* have been shown to act via opioid mechanisms in inducing antinociception action in rats [9, 43]. Therefore, *P.o* may also act via opioid receptors to induce anti-inflammatory activity.

Jedinak et al. [22] showed that water extract of *P.o* suppressed LPS-induced secretion of TNF-*α*, cytokines, and activation of nuclear transcription factor kB which can be related to the mechanism of suppression of carrageenan-induced inflammation by SFDP and AE of *P.o* in the present study.

As evident by the findings of our previous study, *P.o* do not possibly exert any toxic effects in Wistar rats as well as human subjects [14]. Preliminary phytochemical analysis revealed the presence of terpenoids, tannins, steroidal glycosides, and carbohydrates [44].

## 5. Conclusions

The study suggests that culinary mushroom *P.o* possesses anti-inflammatory activity and can be considered as a functional food that has the potential to control inflammation. Antihistamine and membrane stabilizing activities, inhibition of cell migration to the site of inflammation, and inhibition of NO production may be possible mechanisms of anti-inflammatory activity of *P.o.* Anti-inflammatory activity-guided fractionation of AE of *P.o* in the search of potent anti-inflammatory compounds is in progress. We recommend that the potential health benefits of the oyster mushroom be evaluated with clinical studies.

## Data Availability

The data used to support the findings of this study are included within the article.
